# Plant density can increase invertebrate postdispersal seed predation in an experimental grassland community

**DOI:** 10.1002/ece3.2039

**Published:** 2016-05-06

**Authors:** Jan‐Hendrik Dudenhöffer, Gesine Pufal, Christiane Roscher, Alexandra‐Maria Klein

**Affiliations:** ^1^ Nature Conservation and Landscape Ecology Institute of Earth and Environmental Sciences University of Freiburg Tennenbacherstr. 4 79106 Freiburg Germany; ^2^ UFZ Department of Physiological Diversity Helmholtz Centre for Environmental Research Permoserstrasse 15 04318 Leipzig Germany; ^3^ German Centre for Integrative Biodiversity Research (iDiv) Halle‐Jena‐Leipzig Deutscher Platz 5a 04103 Leipzig Germany

**Keywords:** Arthropods, granivory, Janzen–Connell effects, plant species richness, plant–insect interaction, slugs

## Abstract

Janzen–Connell effects are negative effects on the survival of a plant's progeny at high conspecific densities or close to its conspecifics. Although the role of Janzen–Connell effects on the maintenance of plant diversity was frequently studied, only few studies targeted Janzen–Connell effects via postdispersal seed predation in temperate grassland systems. We examined effects of conspecific density (abundance of conspecific adult plants) on postdispersal seed predation by invertebrates of three grassland species (*Centaurea jacea, Geranium pratense, and Knautia arvensis*) in experimental plant communities. Additionally, we examined the impact of plant species richness and different seed predator communities on total and relative seed predation (= seed predation of one plant species relative to others). We offered seeds in an exclusion experiment, where treatments allowed access for (1) arthropods and slugs, (2) arthropods only, (3) small arthropods only, and (4) slugs only. Treatments were placed in plots covering a gradient of abundance of conspecific adults at different levels of plant species richness (1, 2, 3, 4, 8 species). Two of the plant species (*C. jacea and K. arvensis*) experienced higher rates of seed predation and relative predation with increasing abundance of conspecific adults. For *C. jacea*, this effect was mitigated with increasing plant species richness. Differences in seed predator communities shifted seed predation between the plant species and changed the magnitude of seed predation of one plant species relative to the others. We exemplify density‐dependent increase in seed predation via invertebrates in grassland communities shaping both the total magnitude of species‐specific seed predation and seed predation of one species relative to others. Further differences in seed predator groups shift the magnitude of seed predation between different plant species. This highlights the importance of invertebrate seed predation to structure grasslands via density‐dependent effects and differing preferences of consumer groups.

## Introduction

Janzen–Connell effects are hypothesized as a mechanism contributing to the maintenance of plant diversity (Janzen 1970; Connell 1971). Based on the diversity patterns of tropical forests, Janzen–Connell effects originally described an increased mortality of seeds and seedlings due to an accumulation of specialized enemies in the vicinity of their conspecific adults or at high densities of conspecific adults. Since its first introduction, the basic concept of Janzen–Connell effects was extended to a broader range of plant–antagonistic interactions, for example, negative plant‐soil feedback effects due to an accumulation of plant‐specific belowground pathogens over time (Packer and Clay 2000; MacKay and Kotanen 2008; Petermann et al. 2008; Mangan et al. 2010).

In the first meta‐analysis on distance dependent Janzen–Connell effects, Hyatt et al. (2003) found no support for their general validity and suggested that there is no need to further testing. However, a recent meta‐analysis by Comita et al. (2014), which addressed distance‐ and density‐driven Janzen–Connell effects based on a larger number of studies, supports the general validity of both distance‐ and density‐driven Janzen–Connell effects across different biomes and habitats. One prominent cause of Janzen–Connell effects is the predation of seeds by granivorous animals. Seeds present a nutritious resource and are subjected to high predation pressure (Crawley 2000). The destructive consumption of seeds directly kills a part of the plants progeny (Hulme and Benkman 2002) and may heavily reduce the amount of seeds reaching a suitable microsite to germinate (Andersen 1989; Hulme 1998). In particular, in habitats where plant communities are seed limited, seed predation can considerably affect plant community structure (Orrock et al. 2006; Fraser and Madson 2008; Vaz Ferreira et al. 2011).

Notably, Janzen–Connell effects via postdispersal seed predation were only sparsely addressed at the scale of herbaceous plant communities and especially studies in temperate grassland systems still remain underrepresented (Comita et al. 2014). Although some studies examined postdispersal seed predation in temperate experimental and semi‐natural grassland systems, they focused on the effects of plant species richness (Pufal and Klein 2013; Preukschas et al. 2014) and not on the density dependence of seed predation, which would be required to address the evidence for Janzen–Connell effects, that is, an increase in seed predation with increasing abundance of conspecific adults.

In this study, we examined the density dependence of postdispersal seed predation at the scale of local temperate grassland plant communities. In this context, we focus on invertebrate seed predators which are shown to largely contribute to seed predation in agricultural settings (Cromar et al. 1999; Gallandt et al. 2005) and strongly respond at a small spatial scale to plant community characteristics (Pufal and Klein 2013) and the availability of seeds (Frank et al. 2011).

Importantly, the prerequisite that density‐dependent seed predation can contribute to the maintenance of plant diversity differs between seed‐limited and site‐limited plant communities. In a seed‐limited community, the overall number of seeds of one species would directly impact the number of its progeny. Thus, an increased seed predation of that species would reduce its reproductive success. In a site‐limited community, different species compete for a limited number of suitable microsites. Thus, rather the relative contribution of one species to the local seed pool than the total amount of its seeds may determine its chance of occupying those microsites and therefore its reproductive success. In this context, the predation of seeds of one species relative to other species in the community may be of greater importance.

In particular, for the latter case, it is noteworthy that invertebrate seed predators exhibit some degree of seed preferences (Sasakawa 2010; Petit et al. 2014), and different groups are shown to feed on different seed sources. Thus, changes in seed predator communities may cause such species‐specific differences in seed predation with potentially contrasting effects on different plant species on a community level. Moreover, plant species richness (Pufal and Klein 2013; Preukschas et al. 2014) and community characteristics (Russell and Schupp 1998; Meiss et al. 2010) affect the intensities and patterns of postdispersal seed predation with partly differing effects on different seed predator groups (Pufal and Klein 2013, 2015) and thus may interfere with density‐dependent seed predation. Higher plant species richness also entails a higher variety of different seed species. Such increased resource diversity may partly mitigate the adaptation of consumers on the dominant resource and thus the density dependence of seed predation. Following these assumptions, we hypothesize that

1. Species‐specific seed predation increases with increasing abundance of conspecific adult plants.
2. This results in higher relative seed predation of that species compared to other species present in the community.
3. Increasing plant species richness mitigates the density‐dependent seed predation.
4. Different invertebrate seed predator groups cause different seed predation of one species relative to the other species.

To address these hypotheses, we conducted a postdispersal seed predation experiment at the scale of local plant communities in an experimental grassland system, combined with different exclusion treatments to manipulate invertebrate seed predator group communities. Thus, our study investigates for the first time the density dependence of invertebrate postdispersal seed predation in grassland communities.

## Methods

### Study site, experimental setup, and target plant species

We conducted this study within the framework of the ‘Jena‐Experiment’ (Thuringia, Germany; 50°55′ N, 11°35′ E, 130 m a.s.l. (Roscher et al. 2004)). We used experimental plots of varying plant species richness and functional diversity in the so‐called trait‐based diversity experiment (TBE) established in 2010 (Ebeling et al. 2014). The plant communities in the TBE were assembled from 20 Central European mesophilic grass and nonlegume herb species, which were assigned to three species pools representing trait differences related to the dimensions of spatial (i.e., plant height, leaf size, rooting depth, and root length density) and temporal (i.e., start of vegetative growth and onset of flowering) resource acquisition. Plant communities were designed to cover a gradient of plant species richness (1, 2, 3, 4, and 8 sown species) and different levels of functional diversity according to the trait dimension of their respective species pool on a total of 138 plots (3.5 m × 3.5 m). The plant communities were originally sown with equal total density and even proportions of species in the mixtures. The experimental plots are maintained by biannual mowing and three weeding campaigns per year to remove species not sown into a particular plot (see Ebeling et al. 2014 for a detailed description of the experiment).

We chose the three herbaceous perennial species *Centaurea jacea* L., *Knautia arvensis* (L.) Coult., and *Geranium pratense* L. from the species of the TBE as case examples for the seed predation experiment. All three species are common in semi‐natural temperate European grassland systems and exhibit differences in seed traits (see Table 1 for details of seed characteristics). Noteworthy, *C. jacea* and *K. arvensis* belong to the same species pool covering differences in the spatial trait dimension (pool 1) and occurred together in some plots, whereas *G. pratense* belongs to the species pool covering differences in the temporal trait dimension (pool 2) (Ebeling et al. 2014). This entails that the co‐occurring plant species in the communities of *C. jacea* and *K. arvensis* exhibit a relatively similar flowering and seeding phenology (seed dissemination in late summer/autumn), whereas the co‐occurring plant species in the communities of *G. pratense* significantly differ in flowering and seeding phenology (seed dissemination ranges from spring to autumn). We selected all plots where at least one of the three target species was present. This resulted in a total of 37 plots covering all levels of plant species richness and a gradient in the abundance of conspecific adult plants of the target species (see Table S1 in Supplementary Information). The abundance of conspecific adults was estimated as plant cover using a modified scale (Londo 1976). Numerical values for species cover were coded as 0.5 (<1%), 3 (1‐5%) 10 (6‐15%), 20 (16‐25%), 30 (26‐35%), 40 (36‐45%), 50 (46‐55%), 60 (56‐65%), 70 (66‐75%), 80 (76‐85%), and 90 (>85%) in mid‐August. We conducted the experiment in late August/September 2014, at the time of seed dissemination of our target plant species. Seeds for the experiment were purchased from the same commercial supplier that was used for the establishment of the experiment (Rieger‐Hofmann GmbH, Blaufelden‐Raboldshausen, Germany).

**Table 1:** Characteristics of the three seed species used in this study (information on the ranges of seed mass and size were obtained from the BiolFlor database; http://www2.ufz.de/biolflor)

Species	Size (mm)				Appendages	Shape
	Mass (mg)	Length	Width	Diameter		
*Centaurea jacea*	1.9–2.1	2.5–3.5	1.2–1.7	0.8–1.2	Hairs	Ovate
*Knautia arvensis*	4.6–4.7	3.5–6.0	2.0–2.4	1.0–2.0	Hairs, pappus, elaiosome	Oblong
*Geranium pratense*	6.0–8.8	3.0–3.5	1.5–2.0	1.5–2.0	None	Broad elliptic

### Seed predator exclusion treatments

A cafeteria experiment was set up to estimate the effects of different invertebrate seed predator groups on “seed predation rates” and “relative seed predation.” The cafeterias were designed to consecutively exclude invertebrate seed predator groups from access to the seeds. Therefore, we assigned invertebrate seed predators to three groups: large arthropods (LA), small arthropods (SA), and slugs (SL), which were excluded in four exclusion treatments: access for all three invertebrate groups (LA + SA + SL treatment), access for arthropods only (LA+SA treatment), access for small arthropods only (SA treatment), access for slugs only (SL treatment).

**Figure 1:**
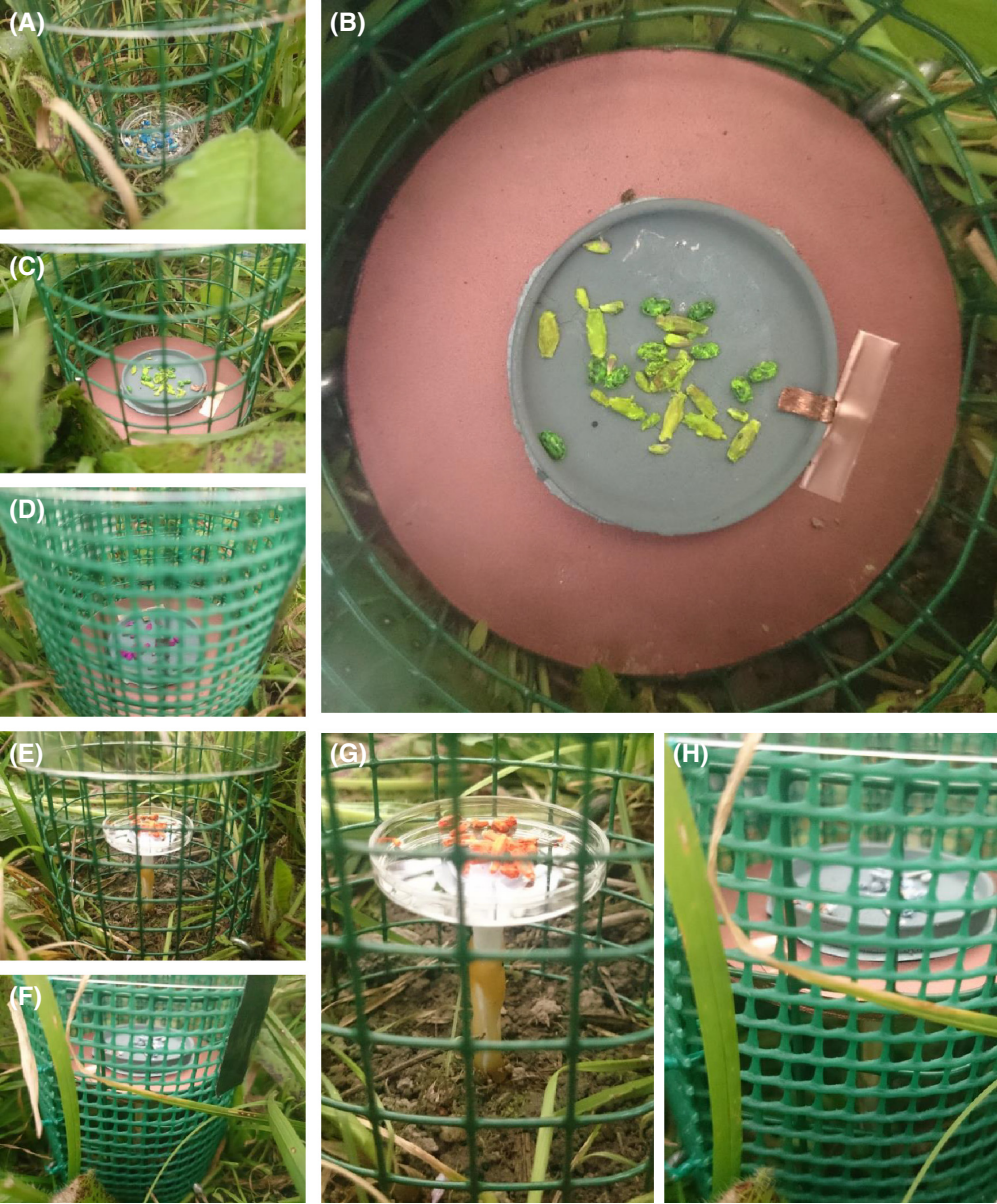
Seed cafeteria exclusion treatments. (A) access for large arthropods, small arthropods, and slugs (LA + SA + SL), (B) access for large arthropods and small arthropods only (LA + SA), (C) access for small arthropods only (SA), (D) access for slugs only (SL), (E) control with all barriers, F: detail on “slug‐exclusion” dish, (G) detail on “slug‐only” dish, (h) detail on the all barriers control.

All treatments consisted of a wire mesh cage (mesh size = 10 mm) with a plastic roof to prevent the access of rodents, birds, and seed dispersal by rain splash. Seeds were placed on plastic dishes (diameter = 38 mm) to prevent access of earthworms (Fig. 1A). This basic setup was used to allow for the access of all invertebrate seed predator groups (= LA + SA + SL treatment).

For the exclusion of slugs, we used an “electrical deterrence” approach, based on the different electrochemical potential of zinc and copper. Therefore, we modified the seed dish as following: The dish was covered with a zinc layer and placed on a copper‐covered base plate. The zinc and copper parts were separated by an isolating barrier and connected at one point by a conductive copper bridge (Fig. 1B). To access the seeds in the dish, slugs had to contact the zinc and the copper parts simultaneously, thereby closing the conductive cycle and inducing an electrical current. In combination with the basic setup, we used the slug‐exclusion dishes for the LA + SA treatment (Fig. 1C).

For the exclusion of large arthropods, we covered the cages of the basic setup with an additional mesh with a mesh size of 3 mm and used them in combination with the slug‐exclusion dishes in the SA treatment (Fig. 1D). In the SL treatment, we placed the seed dish of the basic setup on a golf tie covered with insect glue to prevent access of arthropods (Fig. 1E). We additionally set up a control treatment, excluding all groups (by combining all barriers, Fig. 1F). The offered seeds were color‐coded according to the five exclusion treatments (the four actual exclusions and the control with all barriers) with fluorescent dye (Kremer Pigmente GmbH & Co KG, Aichstetten, Germany) (see Lemke et al. 2009; Pufal and Klein 2013, 2015).

In all plots, we installed one cafeteria of each exclusion treatment. Ten seeds of each of the three target plant species were placed together in every cafeteria dish. After an exposure time of 48 h, remaining seeds were counted. To avoid an overestimation of seed predation rates (see Vander Wall et al. 2005), we searched for missing seeds at night using UV‐flashlights (HWA WYS UltraFire WF‐501B, Wha Fat Technological Co. Ltd, Hong Kong) for 10 minutes per plot and recorded any detected seeds outside the cafeterias (Pufal and Klein 2013, 2015). Detected seeds could be traced back to their exclusion treatment by their color codes. After each observation round, all seeds were removed and replaced with 10 fresh seeds per species to prevent molding or germination of the seeds and to offer an equal amount of seeds before each observation round. In total, we conducted four surveys for each plot. Therefore, we assigned the plots equally to two alternating time blocks shifted by 24 h. All activities related to the survey rounds were adjusted with respect to these time blocks (e.g., day one: placing seeds of block one, day two: placing seeds of block two, day three: counting and nightly searches for seeds of block one, day four: counting and nightly searches for seeds of block two, etc.)

### Statistical analyses

All analyses were performed in R version 3.1.1 (R Core Team, 2014). We calculated seed predation as the net numbers of missing seeds by deducting the numbers of recovered seeds and calculated the response variable “seed predation rate” as *N*_seeds missing_ − *N*_seeds recovered_ as successes versus *N*_seeds provided_ − (*N*_seeds missing_ − *N*_seeds recovered_) as failures (hereafter “seed predation”). Seed predation in the control treatment was only marginal (0.1 ± 2.0%; mean ± SD), indicating the effectiveness of the exclusion barriers. Thus, we restricted the subsequent analyses to the four actual exclusion treatments. For all three target plant species, cover of conspecific adults and plant species richness was only moderately correlated (*r* < 0.2) and variance inflation factors (VIF) indicated no severe problems regarding collinearity after mean centering (VIF < 3). For each of the target species, we tested the fixed effects of the seed predator exclusion treatment (as factor with four levels), the cover of conspecific adults (mean centered), and plant species richness (mean centered) including all 2‐way interactions on the response “seed predation” with generalized linear‐mixed models using binomial error distribution with a logit link function and maximum‐likelihood estimation (R package: lme4, Bates et al. 2014). We accounted for plot identity nested in time block and the survey round nested in time block as random intercepts. To account for overdispersion, we included an observation level random intercept (Elston et al. 2001; Harrison 2014).

To calculate the response variable “relative seed predation,” we pooled the seed losses for each target species over the four observation rounds and calculated “relative seed predation” as *N*_seeds missing target_ − *N*_seeds recovered target_ as successes versus *N*_seeds missing total_ − *N*_seeds recovered total_ as failures. We tested for the above‐mentioned fixed effect structure accounting for plot identity nested in time block as random intercept. Significance of the fixed effects was estimated using Wald type‐II chi‐square tests (R package: car, Fox and Weisberg 2011).

## Results

**Figure 2:**
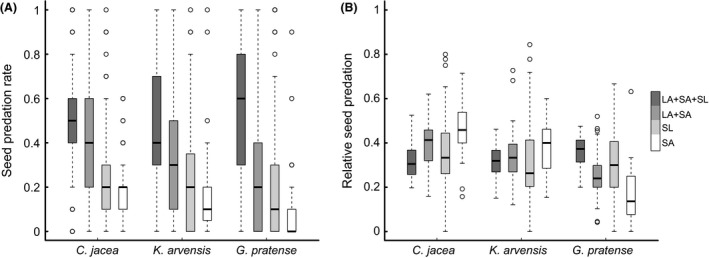
Effects of the four exclusion treatments (LA + SA + SL = access for large arthropods, small arthropods and slugs; LA + SA = access for large arthropods and small arthropods only; SL = access for slugs only; SA = access for small arthropods only) on (A) seed predation rates and (B) relative seed predation of *C. jacea*,* K. arvensis,* and *G. pratense*.

Seed predation of all three target species was significantly affected by the seed predator exclusion treatments (Table 2). Generally, seed predation decreased when more seed predator groups were excluded from access to the seeds. Highest seed predation occurred in the LA + SA + SL treatment followed by the LA + SA treatment, the SL treatment, and the SA treatment (Fig. 2A). The exclusion of seed predator groups also affected relative seed predation of the species (Table 3). The relative predation of *G. pratense* seeds was lower compared to *C. jacea* and *K. arvensis* seeds in the LA+SA and SA treatment (Fig. 2B).

**Table 2:** Binomial generalized linear‐mixed model results for the fixed effects of the exclusion treatments (ET), cover of conspecific adults (CCA), plant species richness (PSR) on seed predation rates of the three target species; significance of fixed effects was estimated using Wald type‐II *χ* ^2^ tests

Fixed effects	df	*Knautia arvensis*		*Centaurea jacea*		*Geranium pratense*	
		***χ*** ^**2**^	*P*	***χ*** ^**2**^	*P*	***χ*** ^**2**^	*P*
Exclusion treatment (ET)	3	235.82	**<0.001**	249.16	**<0.001**	274.86	**<0.001**
Cover of conspecific adults (CCA)	1	66.34	**<0.001**	22.56	**<0.001**	4.51	**<0.05**
Plant species richness (PSR)	1	3.05	0.081	6.28	**<0.05**	0.30	0.584
ET × CCA	3	33.62	**<0.001**	33.36	**<0.001**	6.34	0.096
ET × PSR	3	4.52	0.210	1.46	0.693	8.43	**<0.05**
CCA × PSR	1	3.13	0.077	21.41	**<0.001**	1.34	0.248

Significant effects (*P* < 0.05) are highlighted in bold.

**Table 3:** Binomial generalized linear‐mixed model results for the fixed effects of the exclusion treatments (ET), cover of conspecific adults (CCA), plant species richness (PSR) on the relative seed predation of the three target species; significance of fixed effects was estimated using Wald type‐II *χ* ^2^ tests

Fixed effects	df	*Knautia arvensis*		*Centaurea jacea*		*Geranium pratense*	
		*χ* ^2^	*P*	*χ* ^2^	*P*	*χ* ^2^	*P*
Exclusion treatment (ET)	3	18.75	**<0.001**	41.27	**<0.001**	20.86	**<0.001**
Cover of conspecific adults (CCA)	1	11.79	**<0.001**	3.12	0.077	0.21	0.650
Plant species richness (PSR)	1	0.26	0.610	0.31	0.577	1.50	0.221
ET × CCA	3	8.87	**<0.05**	5.16	0.160	0.63	0.889
ET × PSR	3	0.15	0.985	3.08	0.379	1.11	0.774
CCA × PSR	1	3.09	0.079	6.85	**<0.01**	0.07	0.791

Significant effects (*P* < 0.05) are highlighted in bold.

**Figure 3:**
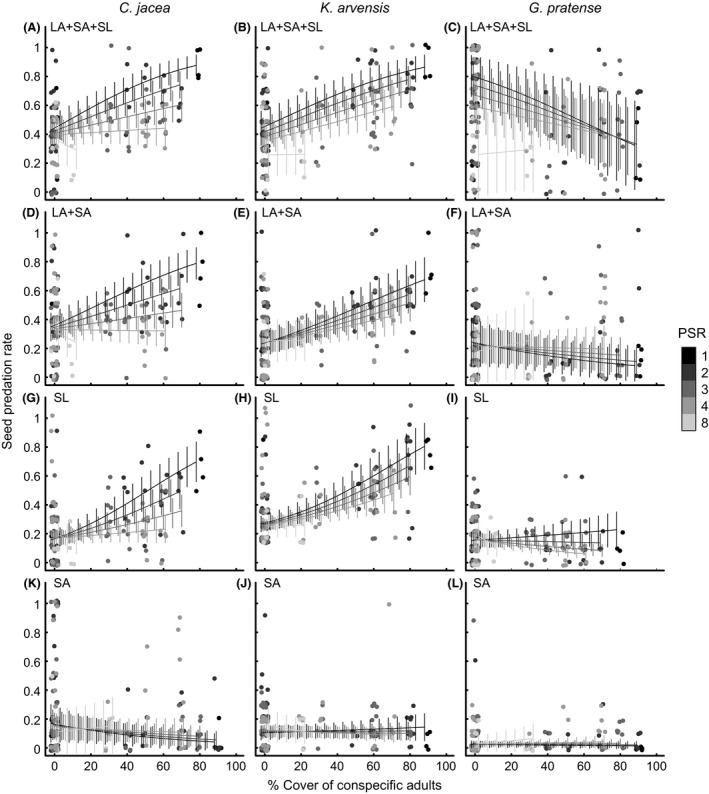
Effects of cover of conspecific adults and plant species richness (PSR) on seed predation rates of *C. jacea*,* K. arvensis,* and *G. pratense* in the four exclusion treatments (LA + SA + SL = access for large arthropods, small arthropods and slugs; LA + SA = access for large arthropods and small arthropods only; SL = access for slugs only; SA = access for small arthropods only); vertical lines denote 2 SE of the regression fits; points are slightly jittered in *y*‐axis direction to improve visualization.

In all but the SA treatment, predation of *C. jacea* and *K. arvensis* seeds increased with increasing abundance of their conspecific adults (Fig. 3A,B,D,E,G,H). For *C. jacea*, this effect was moderated by plant species richness of the communities (Table 2) and mitigated at higher levels of plant species richness (Fig. 3A,D,G). In contrast, seed predation rates of *G. pratense* decreased with increasing abundance of its conspecific adults in the LA+SA+SL treatment and, less pronounced, in the LA+SA treatment (Fig. 3C,F).

**Figure 4:**
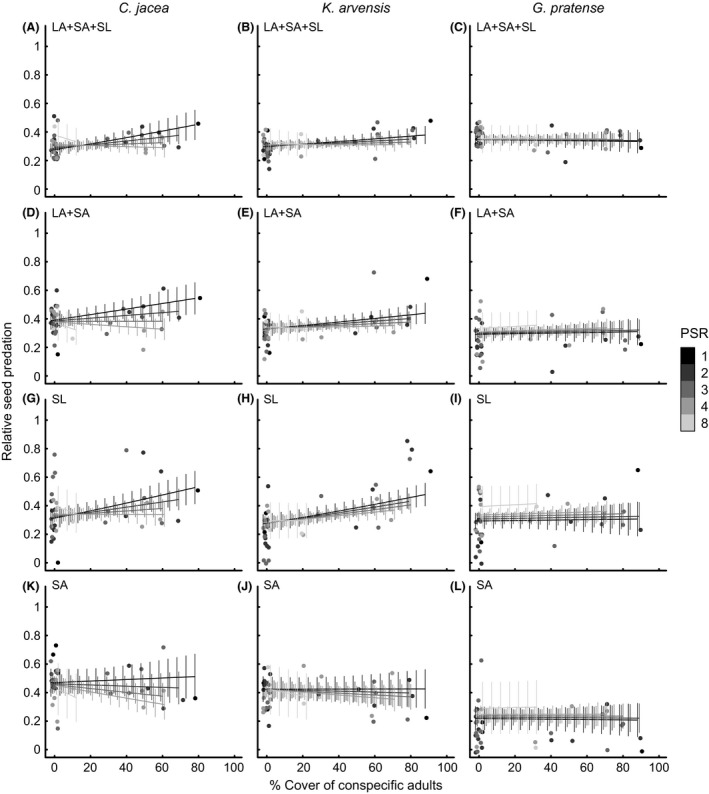
Effects of cover of conspecific adults and plant species richness (PSR) on relative seed predation of *C. jacea*,* K. arvensis,* and *G. pratense* in the four exclusion treatments (LA + SA + SL = access for large arthropods, small arthropods and slugs; LA + SA = access for large arthropods and small arthropods only; SL = access for slugs only; SA = access for small arthropods only); vertical lines denote 2 SE of the regression fits; points are slightly jittered in y‐axis direction to improve visualization.

Similar to the seed predation, the relative seed predation of *C. jacea* and *K. arvensis* also increased with increasing abundance of their conspecific adults (Fig. 4A,B,D,E,G,H). Again, for *C. jacea,* this effect was mitigated with increasing plant species richness (Table 3; Fig. 4A,D,G). The relative seed predation of *G. pratense* was not affected by the abundance of conspecific adults or by plant species richness (Table 3, Fig. 4C,F,I). Even if the plots of the highest species‐richness level with eight species (with a lower number of replicates) were excluded from the analyses, results did not change qualitatively (Tables S2 and S3 in Supporting Information).

## Discussion

### Differences in species‐specific seed predation patterns and density dependence

In contrast to the consistent patterns of increasing seed predation with increasing abundance of conspecific adults found for *C. jacea* and *K. arvensis*, predation of the seeds of *G. pratense* decreased with increasing abundance of conspecific adults, particularly in the treatments where slugs had access (LA+SA+SL and SL). A food choice experiment showed that leaves of *G. pratense* are not attractive for slugs (Kozłowski and Kozłowska 2004). Therefore, we assume that overall slug feeding activity, and thus the probability to encounter the offered seeds, is lower in plots where *G. pratense* plants are present. However, seed predation of *G. pratense* relative to those of *C. jacea* and *K. arvensis* was generally lower in the treatments were slugs were excluded (LA + SA and SA). This indicates that seeds of *G. pratense* may be attractive for slugs, potentially because of the lacking appendages, for example hairs, which makes it easier for slugs to swallow the entire seed. Compared to the other two species, *G. pratense* seeds possess a hard seed coat (Meisert 2002), which probably makes them unattractive to at least arthropod seed predators. Notably, *G. pratense* also belongs to the species pool that was designed to cover the temporal trait dimension, which includes, for example, the onset of flowering. In this case, availability of seed resources at the given time point might be more variable between communities of different species compositions, although we conducted the experiment at the time of seed dissemination of the target species. In contrast, the species in the communities of *C. jacea* and *K. arvensis* exhibit a more similar phenology. Thus, we assume that the overall abundance of seed resources is higher in the latter communities making them generally more attractive for seed predators.

In the SA treatment, we excluded all animals with a size above 3 mm. We observed ants as the predominant group with access to the seeds in that case (author's personal observations). Beside the overall lowest magnitude in the SA treatment, predation occurred predominantly on the small‐sized seeds of *C. jacea* and on the seeds of *K. arvensis,* which bear elaiosomes (fatty appendages) and are known to be attractive for ants (Retana et al. 2004). This might also explain the lack of density dependence of seed predation, as other factors, for example, the distance of the offered seeds to the next ant colony might be of greater importance (Diaz 1992).

### Effects of plant species richness on seed predation

In contrast to studies showing that seed predation increased with increasing plant species richness or diversity (Vockenhuber et al. 2013; Preukschas et al. 2014), we found no such relation in our experiment. Common explanations for increased predation pressure at higher plant diversity are positive bottom‐up effects of plant diversity on the diversity and abundance of consumers, which leads to an increased resource exploitation. However, the strength of bottom‐up effects depends on the trophic level of the consumers, with only weak direct effects on omnivores (Scherber et al. 2010). Moreover, the relation of consumer diversity and resource exploitation applies to specialized consumers (Finke and Snyder 2008) and high consumer densities (Griffin et al. 2008). In temperate climates, postdispersal seed predation by invertebrates is less dependent on specialized consumers and generally driven by a diverse consumer community (Lundgren et al. 2013) of mostly omnivorous groups (Honěk et al. 2003; Saska 2008; Koprdová et al. 2010). Hence, direct bottom‐up effects of plant species richness on specialized seed predators are probably not an important driver for the magnitude of seed predation, especially in the context of our study with its relatively low levels of plant species richness.

In contrast, for *C. jacea,* the increase in seed predation and relative seed predation with increasing abundance of conspecific adults was mitigated with increasing plant species richness. Despite the low level of specialization of seed predators, they strongly respond to the presence of seed resources by adapting their feeding behavior with respect to resource availability and abundance (Frank et al. 2011). They still exhibit some degree of seed preferences related to seed traits (Honěk et al. 2007; Lundgren and Rosentrater 2007) and the distribution of seed resources (Marino et al. 2005). Increased plant species richness is directly linked with a higher diversity of seed resources and thus may foster a broader variety of feeding preferences which leads to more even predation of different seeds relaxing the predation pressure on a single species. However, as the mitigating effect of plant species richness was only evident for one of our target species, we can hardly draw conclusions of its general validity.

### Impact of seed predation and density dependence on plant communities

There is a considerable debate on the importance of seed limitation for plant community composition and dynamics (Andersen 1989; Clark et al. 2007; Duncan et al. 2009). However, there is strong evidence that seed limitation impacts plant recruitment and thus community composition (Fraser and Madson 2008; Stein et al. 2008; Orrock et al. 2009; Russell et al. 2010). Seed predation can be an important driver of seed limitation and thus the local patterns of community composition and plant abundance (Orrock et al. 2006; Vaz Ferreira et al. 2011).

In our system, an experimental grassland community, it was repeatedly shown that seed addition caused significant changes in plant community compositions (Roscher et al. 2009, 2014; Petermann et al. 2010), suggesting that density‐driven seed predation and differential seed predation of seed predator groups are important factors for the structuring of plant communities in this system. Moreover, a surplus of seeds resulted in even stronger effects on the plant communities at low levels of species richness. Remarkably, also density‐dependent seed predation of *C. jacea* was even stronger at low levels of plant species richness. If density‐dependent seed predation is more pronounced in plant communities of low species richness which are more susceptible to seed addition, this would indicate a self‐stabilizing mechanism of plant diversity.

However, even for the extreme case that plant communities are purely limited by the availability of suitable sites, species‐specific seed losses and the magnitude at which seed predation occurs on different plant species can influence plant community dynamics. In a situation where different plant species compete for a limited number of free sites suitable for germination, the chance that a sufficient number of seeds of a certain species reaches and occupies those sites increases with its proportion of the total seed pool. Uneven seed loss between plant species may not affect whether a suitable site gets occupied but can determine the identity of the occupying species. We observed such an increased relative seed predation as result of increasing density of conspecific adults for *C. jacea* and *K. arvensis*. Consequently, this lowers their contribution to the local seed pool when becoming more abundant. Such changes in the relative composition of the seed pool may interfere with stochastic assembly processes from the local species pool (Tofts and Silvertown 2002; Germain et al. 2013).

Even those plant species with large and long‐lived seeds were shown to benefit from increased seed supply (Roscher et al. 2009) and are especially prone for postdispersal seed predation (Clark et al. 2007). So differences in species‐specific seed predation may cascade through time and become more important at a longer timescale if long‐lived seeds accumulate in the seed bank. In particular, after disturbance events, the relative contribution of a plant species to the local seed bank may determine its future performance (Maron and Gardner 2000).

## Conclusion

In this study, we exemplified density‐driven Janzen–Connell effects via postdispersal seed predation for two of our three target species in a temperate grassland system. While these results partly support the evidence of Janzen–Connell effects in this system, they also indicate that the significance of this mechanism may be different depending on plant species identity. Together with the increase in seed predation with increasing abundance of conspecific adults, we found also an increasing relative predation of seeds of the respective plant species, which supports, that Janzen–Connell effects may contribute to the maintenance of diversity in both seed‐ and site‐limited plant communities. Additionally, we show that differences in seed predator groups caused differential seed predation of the three species, indicating that seed predator communities may play an important role shaping the reproductive success of one species relative to other species in a community. This study supports our hypothesis that postdispersal seed predation can be density‐dependent and can therefore be important to structure grassland communities. Our study identifies for the first time Janzen–Connell effects via postdispersal seed predation in a temperate grassland system with likely consequences for plant reproduction. This implies that grassland management should consider plant species‐specific densities in seed mixtures for grassland conservation and restoration, especially when plant species sensitive to postdispersal seed predation are included.

## Conflict of Interest

None declared.

## Supporting information


**Table S1.** Summary of the experimental plots used in this study varying in plant species richness (PSR) and abundance of conspecific adults of the target species (Cover).
**Table S2.** Binomial generalized linear mixed model results for the fixed effects of the exclusion treatments (ET), cover of conspecific adults (CCA), plant species richness (PSR) on seed predation of the three target species when the eight species mixtures were excluded from the analyses; significance of fixed effects was estimated using Wald type II χ^2^ tests.
**Table S3.** Binomial generalized linear mixed model results for the fixed effects of the exclusion treatments (ET), cover of conspecific adults (CCA), plant species richness (PSR) on the relative seed predation of the three target species when the 8 species mixtures were excluded from the analyses; significance of fixed effects was estimated using Wald type II χ^2^ tests.
