# Supplementary material for: Plant density can increase invertebrate postdispersal seed predation in an experimental grassland community
Source: Ecol Evol. 2016 May 6;6(11):3796–807. doi: 10.1002/ece3.2039 (PMC4864194; doi:10.1002/ece3.2039)
Supplement: Supplementary file 1 — Table S1. Summary of the experimental plots used in this study varying in plant species richness (PSR) and abundance of conspecific adults of the target species (Cover). Table S2. Binomial generalized linear mixed model results for the fixed effects of the exclusion treatments (ET), cover of conspecific adults (CCA), plant species richness (PSR) on seed predation of the three target species when the eight species mixtures were excluded from the analyses; significance of fixed effects was estimated using Wald type II χ2 tests. Table S3. Binomial generalized linear mixed model results for the fixed effects of the exclusion treatments (ET), cover of conspecific adults (CCA), plant species richness (PSR) on the relative seed predation of the three target species when the 8 species mixtures were excluded from the analyses; significance of fixed effects was estimated using Wald type II χ2 tests. [file ECE3-6-3796-s001.docx]

**SUPPORTING INFORMATION**

**Table S1.** Summary of the experimental plots used in this study varying in plant species richness (PSR) and abundance of conspecific adults of the target species (Cover).

**Plot PSR Cover *_C.jacea_* Cover *_K. arvensis_* Cover *_G. pratense_***

1 3 40 0 0

7 2 0 0 50

10 4 50 30 0

13 2 70 0 0

17 3 0 0 40

18 8 0 0 30

20 1 0 90 0

27 1 80 0 0

30 4 50 20 0

33 3 0 60 0

35 4 0 0 30

42 2 40 50 0

53 3 30 60 0

57 4 60 0 0

73 8 10 20 0

74 2 50 0 0

81 2 0 0 40

82 3 60 0 0

91 2 0 0 70

94 3 30 30 0

95 3 0 0 70

99 2 0 60 0

100 2 0 80 0

102 2 0 0 90

103 4 0 70 0

109 4 0 0 50

111 4 0 0 70

113 3 50 0 0

119 4 0 60 0

120 2 60 0 0

121 1 0 0 90

128 3 0 0 80

129 3 0 80 0

130 3 0 0 80

135 2 0 80 0

138 3 0 60 0

**Table S2.** Binomial generalized linear mixed model results for the fixed effects of the exclusion treatments (ET), cover of conspecific adults (CCA), plant species richness (PSR) on seed predation of the three target species when the 8 species mixtures were excluded from the analyses; significance of fixed effects was estimated using Wald type II χ^2^ tests

***K. arvensis C. jacea G. pratense***

**Fixed effects d.f. χ^2^ p χ^2^ p χ^2^ p**

Exclusion treatment (ET) 3 225.26 **<0.001** 237.90 **<0.001** 266.78 **<0.001**

Cover of conspecific adults (CCA) 1 64.80 **<0.001** 23.74 **<0.001** 4.56 **<0.05**

Plant species richness (PSR) 1 0.77 0.38 3.30 0.069 0.69 0.405

ET x CCA 3 33.66 **<0.001** 34.77 **<0.001** 6.45 0.092

ET x PSR 3 5.10 0.165 5.48 0.140 6.11 0.106

CCA x PSR 1 3.40 0.065 22.56 **<0.001** 0.09 0.340

**Table S3.** Binomial generalized linear mixed model results for the fixed effects of the exclusion treatments (ET), cover of conspecific adults (CCA), plant species richness (PSR) on the relative seed predation of the three target species when the 8 species mixtures were excluded from the analyses; significance of fixed effects was estimated using Wald type II χ^2^ tests

***K. arvensis C. jacea G. pratense***

**Fixed effects d.f. χ^2^ p χ^2^ p χ^2^ p**

Exclusion treatment (ET) 3 17.77 **<0.001** 43.31 **<0.001** 19.87 **<0.001**

Cover of conspecific adults (CCA) 1 11.71 **<0.001** 3.08 0.079 0.16 0.689

Plant species richness (PSR) 1 1.26 0.262 1.16 0.281 2.95 0.086

ET x CCA 3 8.96 **<0.05** 5.60 0.133 0.52 0.892

ET x PSR 3 1.17 0.759 1.91 0.590 2.11 0.540

CCA x PSR 1 2.07 0.151 4.89 **<0.05** 0.03 0.859
